# Prevalence and risk factors of elevated blood lead levels in 0-6-year-old children: a national cross-sectional study in China

**DOI:** 10.3389/fpubh.2025.1546842

**Published:** 2025-03-26

**Authors:** Min-Ming Li, Xian-Ting Jiao, Jing Zhang, Zhen-Yan Gao, Jia Cao, Jun-Xia Liu, Yu-Lin Yang, Chong-Huai Yan

**Affiliations:** ^1^Children’s Health Department, Shanghai Center for Women and Children’s Health, Shanghai, China; ^2^Ministry of Education-Shanghai Key Laboratory of Children’s Environmental Health, Xinhua Hospital Affiliated to Shanghai Jiao Tong University School of Medicine, Shanghai, China; ^3^Department of Pediatric Infectious, Xinhua Hospital Affiliated to Shanghai Jiao Tong University School of Medicine, Shanghai, China; ^4^The Women’s and Children’s Department, Shanghai Key Laboratory of Maternal Fetal Medicine, Shanghai First Maternity and Infant Hospital, School of Medicine, Tongji University, Shanghai, China

**Keywords:** children, elevated blood lead levels, prevalence, risk factors, China

## Abstract

**Aims:**

To evaluate the prevalence and risk factors of elevated blood lead levels (EBLL) among the pediatric population in China.

**Methods:**

Questionnaire investigation about Lead exposure information, venous blood samples collection and BLL detection are conducted. A total of 32,543 subjects aged 0–6 years old (from 1 month old to under 7 years old) were recruited from May 2013 to March 2015 in 15 provinces of China.

**Results:**

The overall weighted prevalence of EBLL which is defined as BLL ≥ 50 μg/L in this study is 4.1%, as for different geographical regions, with lowest prevalence in the western region of China, lowest prevalence in Shaanxi province and highest in Hebei province. In 0–3-Year-old children, female weighted prevalence of EBLL (4.0%) is higher than male (2.4%), while in 3–6-Year-old children, male (8.3%) is higher than female (6.3%). Bad hygienic habits, some kind of custom, using folk prescriptions, living on the ground floor, poor drinking water quality, indoor air pollution and passive smoking exposure remain risk factors of EBLL (BLL ≥ 50 μg/L) of 0–6-year-old (from 1 month old to under 7 years old) children in China, after adjustment of gender, age, geographical region, annual household income, educational background and occupation of the parents and caregivers.

**Conclusion:**

This study reveals the prevalence and risk factors for EBLL (BLL ≥ 50 μg/L) in 0–6-Year-old Children of China. We hope this study will help public health education and inform policy for preventing and eradicating children’s lead poisoning in China.

## 1 Introduction

Lead naturally occurs in the earth’s crust and has been used in industry, paint, gasoline, construction, pottery, folk medicines, and herbal remedies for centuries. Lead is pervasively present in the environment, slowly impacting health from early childhood and throughout life (1). When lead is absorbed in different ways, it enters the body, attaches to soft tissue, easily assimilated into the nervous system (2). Lead poisoning causes toxicity in several organ systems, such as central nervous system impairment what needed special attention, mainly manifested as impairing neurological development, reducing intelligence quotient, and generating negative behavior (3, 4). The adverse effects of childhood lead exposure have been well-documented, such as learning issues (5), behavior problems (6, 7), worsened cognition (8, 9), and the like (10–12). Even blood lead levels less than 50 μg/L are associated with irreversible neurocognitive and behavioral development impairments in infants and children who are more susceptible to lead toxicity (13, 14).

Lead poisoning has been recognized for centuries for its extensive application and environmental persistence (1, 13); however, lead exposure is still a critical public health problem globally, especially in developing countries (15), as poverty may lead to significant ongoing exposures (16).

As a developing country, China has experienced dramatic industrial and economic growth over the past decades, becoming one of the largest lead producers and consumers worldwide. Different sources of children lead exposure are reported in different countries affected by the economic, social and cultural levels. Children still face risks of lead exposure from coal burning, petroleum fuel consumption, e-waste recycling piles (17–19), ore and metal processing, lead-containing folk remedies (20–22), and poor living environment (19, 23–26).

In 2006 public guideline for the prevention of high BLLs and lead poisoning in children issued by the Ministry of Health in China stated that high blood lead syndrome in children should be recognized if the venous blood lead level reaches 100–199 μg/L two consecutive times. Furthermore, lead poisoning in children should be diagnosed if venous blood lead level is ≥200 μg/L two consecutive times (27). Since leaded gasoline was prohibited in 2000 in China, the children BLLs and the prevalence of lead poisoning have dramatically declined over the past 20 years (28, 29). However, the American Centers for Disease Control reports that there is no acceptable level of lead in the human body and that even minimal levels of lead can cause neurological damage to children (30). The Council of State and Territorial Epidemiologists’(CSTE) blood lead reference value is 3.5 μg/dL which is used to identify adults and children whose blood lead levels are higher than the 97.5th percentile of adults and children nationwide (30). Considering sensitivity to lead toxicity in children’s neurocognitive and behavioral development, even BLLs less than 50 μg/L, we defined BLL ≥ 50 μg/L as EBLL in this study, referring to acceptable children’s blood lead levels in 2012 by the American Centers for Disease Control.

For the lack of national epidemiological investigation of blood lead levels and Pb exposure risk factors of 0–6-Year-old children (from 1 month old to under 7 years old) in China, we conduct this national population-based cross-sectional survey. This study explores the prevalence of EBLL, defined as BLL ≥ 50 μg/L, and the risk factors of EBLL. We aim to reveal the epidemiological characteristics of EBLL and provide a theoretical basis for targeted preventive measures of lead exposure for children.

## 2 Materials and methods

### 2.1 Study design

The study population is selected using a multi-stage stratified cluster random sampling technique. The survey methods and sampling details have been published elsewhere (31). The sampling steps are as follows: First, every five provinces were selected from Eastern China, Central China and Western China by the simple random sampling (SRS) method. According to the classification by the National Bureau of Statistics, Shanghai, Jiangsu, Hebei, Guangdong, and Fujian are categorized as eastern regions; Shanxi, Henan, Hunan, Jilin, and Hubei as central regions; and Shaanxi, Yunnan, Qinghai, Guangxi, Xinjiang as western regions. Second, within each selected province, a probabilistic proportional to size (PPS) sampling method was used to choose districts of major cities, medium and small cities and towns/villages of rural areas based on the population. According to the “China Small and Medium City Development Report (63)” and the “Regulations on the Statistical Division of Urban and Rural Areas,” cities with a resident population of over 1 million are classified as major cities, while those with a resident population of 1 million or less are categorized as small and medium-sized cities. Areas outside these designated urban regions are defined as rural areas. Third, children from 1 to 2 neighborhoods chosen by the SRS method within each selected district/village were investigated.

### 2.2 Sample size calculation

The study uses a multi-stage stratified cluster random sampling method. The formula $n=\frac{Z^{2}\cdot P\cdot \left(1-P\right)}{d^{2}}\times \mathrm{deff}$ is used to calculate the sample size for each stratification, in which as for 95% CI, Z sets to 1.96, *p* as expected prevalence rate set to 6%, based on the results of we previous meta-analysis that the pool lead poisoning rate of Chinese population aged 0–18 years old was 5.3% and the pool lead poisoning rate of children was from 4.7 to 9.5% of different age groups under 7 years old (excluding newborn) (29). The allowable error d setting to 2% is applied here. Design effect deff is applied when considering representativeness of 15 provinces of three regions (i.e., Eastern China, Central China, Western China), rural and urban areas (i.e., major cities, medium and small cities and rural areas), gender (i.e., male and female) and age (i.e., 0–36 months and 36–84 months) which are the stratified analysis factors. Consequently, this calculation resulted in a sample size of 24,390 Taking response rate of 90% into account, the final sample size was targeted at 27100. At last, there were over 30,000 samples collected.

### 2.3 Data collection procedure

The survey is strictly quality-controlled. All investigators undertaken by experienced medical professional, are well trained by project team to ensure using standardized questionnaire instructions, standard sample collection, preservation and transportation. Each questionnaire undergoes double scanning and entry, with 10% of the questionnaires being reviewed by the project quality control officers. We obtain sociodemographic information (gender, date of birth, nationality, education level, occupation, and annual family income), self-reported behavior tendencies (smoking, physical activity, hand-to-mouth activity, and washing hands before eating), and living environment for estimating potential lead exposure (folk prescriptions for treatment, customs and habits leading to potential lead exposure, industry around the house, and house location). We obtain the information through face-to-face interviews using a questionnaire of mostly closed-ended questions. We survey Uyghur participants jointly with a Uyghur language translator using a questionnaire translated into Uyghur language. The participants’ confidentiality and privacy are assured. After completion of the interview, anthropometric measurements and blood samples are obtained.

### 2.4 Blood sample collection and detection

A trained nurse drew 3 milliliters of venous blood into a vacuum blood tube. EDTA (Na_2_) is used as an anticoagulant, and samples were stored at 4°C. Refrigerated batches of 100 tubes are sent in a box of ice to the central laboratory at the local site and stored at −20°C before measurement. Blood sample collection is performed in a clean room, far from pollution sources. Children are required to clean their hands with soap before blood collection. Blood lead concentration is measured using an atomic absorption spectrometry-graphite furnace (PerkinElmer Company, 900Z, United States). Quality control is performed using a sample test. The laboratory protocol includes daily calibration with five standards (1–50 μg/L; agreement <5%) and standard reference materials (Contox, Kaulson Laboratories, Inc., NJ, United States) to ensure the accuracy of the assay. The method’s limit of detection (LOD) is 1 ug/dL for BLL. No sample has a BLL less than the LOD. All plastic tubes used for blood lead tests are washed thoroughly, soaked with dilute nitric acid, rinsed with deionized water, and dried before use.

### 2.5 Ethics approval

The study is approved by Ethics Committee of Xinhua Hospital affiliated with Shanghai Jiao Tong University School of Medicine (XHEC-C-2013-008). Signed consent forms are collected from the guardians of all children who participated in the study.

### 2.6 Statistical analysis

Data weighting is used as a statistical technique to adjust survey data to represent the target population. The design weight for adjusting the probability of being sampled for each individual unit of the smallest survey unit is computed by multiplying the sampling weight of each stage. As describing in study design section of this article, we applied three sampling steps and stratified sampling which divided the sample into subgroups as below: different provinces, urban areas and rural areas, different genders and different age groups. The formula $\mathrm{Wh}=\frac{N_{h}/N}{n_{h}/n}$ is applied. N in formula is the total population, Nh represents the number of people in layer h of the population, n is the sample population, n_h_ represents the population of layer h in the sample. We adjust the design weight using the no-answer factor, which is the reciprocal of the response rate. Given the known stratification structure of the population (such as urban and rural areas, age, gender, etc.), the sample is re-stratified and the weights of each stratum are adjusted to match the overall proportion. Population information refers to the data from The Sixth National Population Census of China in 2010. In this survey, the post-stratification weights were adjusted according to age and gender stratification.

Sample characteristics are summarized using descriptive statistics (n, mean, median, p25, p75). A threshold of 50 μg/L for BLLs is used to categorize BLLs into two groups. The socioeconomic status, residence characteristics, and potential risk factors for lead exposure among children are compared between different BLL groups. A logistic regression analysis is performed to estimate odds ratios (OR), 95% confidence intervals (CI), and *p*-values. IBM SPSS version 22 is used for statistical analysis. Statistical significance is set at a *p*-value of 0.05 (two-tailed test).

## 3 Results

This population-based cross-sectional study is conducted to examine the prevalence and associated risk factors of EBLL (BLL ≥ 50 μg/L) among Chinese children aged 0–6 years old (from 1 month old to under 7 years old), and their blood samples are also obtained. We finally recruited 32,543 0–6-year-old children (from 1 month old to under 7 years old) from 15 provinces of China from May 2013 to March 2015 with 16,997 (52.2%) males and 15,546 (47.8%) females.

### 3.1 The prevalence of children with BLL ≥ 50 μg/L (EBLL) in different geographical regions of China

The weighted prevalence with BLL ≥50 μg/L (EBLL) in 0–6-year-old children (from 1 month old to under 7 years old) is 4.1%. There is significant difference of prevalence among different geographical regions of China with 2.7% in the western region much lower than the other two regions in China.

Considering geographic factor, children in rural areas of eastern (12.4%) and western (7.4%) regions have higher prevalence of EBLL than urban areas, while children in urban areas of middle or small-sized city in central region (7.4%) have higher prevalence of EBLL.

**Figure 1:**
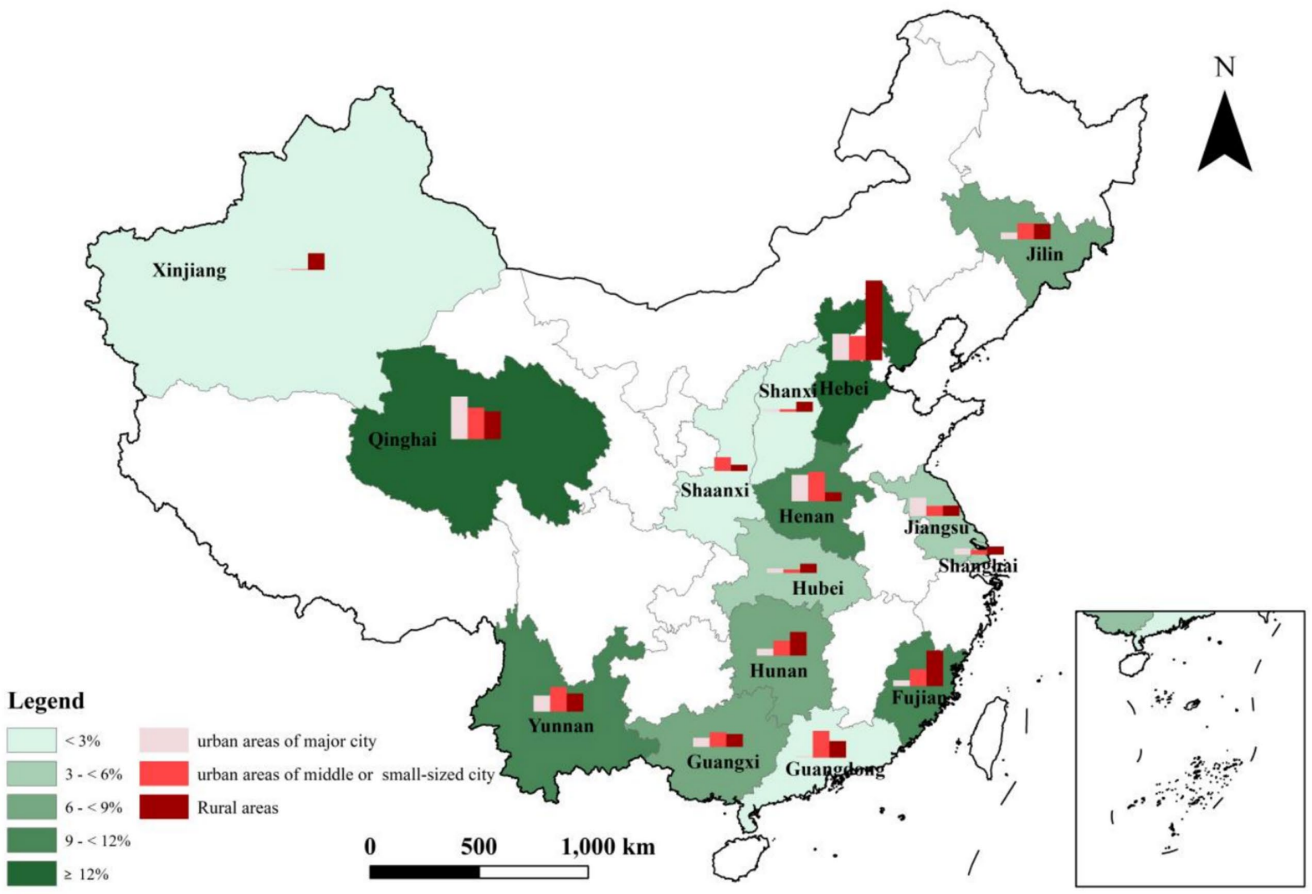
Distribution and EBLL prevalence in 0–6-year-old children of fifteen provinces in China.

And prevalence of EBLL differs significantly by province, with the lowest in Shaanxi province (0.5%) and highest in Hebei province (18.5%) (Figure 1). There is significant difference in children EBLL prevalence of urban areas in major cities (1.8%), urban areas in medium and small cities (7.2%) and in rural areas (8.5%). However, there are significant differences in prevalence among different districts in some provinces. In Qinghai, highest prevalence of urban areas in major city (21.9%) and in middle or small-sized city (16.2%) are observed. In Hebei, highest prevalence of rural areas (41%) is observed (Table 1).

**Table 1:** The EBLL prevalence of children in different geographical regions of China.

Geographical region	Province	*N* (%)	Weighted prevalence of province (%)	Different districts						
				*N* (%)	Weighted prevalence of urban areas of major city (%)	*N* (%)	Weighted prevalence of urban areas of middle or small-sized city (%)	*N* (%)	Weighted prevalence of rural areas (%)	*p*
Eastern region	Shanghai	2,255 (6.9)	3.1	680 (30.2)	3.1	792 (35.1)	2.4	783 (34.7)	4.1	0
	Jiangsu	3,257 (10.0)	6	1,274 (39.1)	9.4	806 (24.7)	5.2	1,177 (36.1)	5.3	0
	Hebei	1,492 (4.6)	18.5	506 (33.9)	13.6	716 (48)	12.5	270 (18.1)	41	0
	Guangdong	1,791 (5.5)	2.8	150 (8.4)	1	782 (43.7)	13.8	859 (48)	8.6	0
	Fujian	1,977 (6.1)	9.3	575 (29.1)	3	708 (35.8)	8.6	694 (35.1)	18.2	0
	total of Eastern region	10,772 (33.1)	5.2*	3,185 (29.6)	2.6	3,804 (35.3)	7.8	3,783	12.4 (35.1)	0
Central region	Shanxi	2,554 (7.8)	2.8	772 (30.2)	1.4	849 (33.2)	1.3	933 (36.5)	5.1	0
	Henan	2095 (6.4)	10.3	622 (29.7)	13.6	712 (34)	15.1	761 (36.3)	4.6	0
	Jilin	2,140 (6.6)	6.5	702 (32.8)	3.4	350 (16.4)	8.2	1,088 (50.8)	7.9	0
	Hunan	2,314 (7.1)	7	697 (30.1)	3.6	940 (40.6)	7.7	677 (29.3)	12.2	0
	Hubei	2,269 (7.0)	3.1	716 (31.6)	2.3	730 (32.2)	1.8	823 (36.3)	4.8	0
	Total of Central region	11,372 (34.9)	6.1*	3,509 (30.9)	5	3,581 (31.5)	7.4	4,282 (37.7)	5.9	0
Western region	Shaanxi	1,681 (5.2)	0.5	309 (18.4)	0	625 (37.2)	7.1	747 (44.4)	3.2	0
	Yunnan	2,241 (6.9)	10.3	639 (28.5)	8.2	840 (37.5)	12.6	762 (34)	9.3	0
	Qinghai	2,465 (7.6)	17.2	882 (35.8)	21.9	779 (31.6)	16.2	804 (32.6)	14.2	0
	Guangxi	1,936 (5.9)	6.2	789 (40.8)	4.7	593 (30.6)	7.5	554 (28.6)	6.6	0
	Xinjiang	2,076 (6.4)	2.5	886 (42.7)	0.6	104 (5)	0.3	1,086 (52.3)	8.5	0
	Total of Western region	10,399 (32.0)	2.7*	3,505 (33.7)	1	2,941 (28.3)	6.5	3,953 (38)	7.4	0
Total		32,543 (100)	4.1	10,199 (31.3)	1.8	10,326 (31.7)	7.2	12,018 (36.9)	8.5	0

* Means *p* < 0.05.

### 3.2 The prevalence of children with BLL ≥ 50 μg/L (EBLL) by different ages and genders

The weighted prevalence of children with BLL ≥50 μg/L (EBLL) is higher in female (4.9%) than in male (3.7%). However, the situation varies among different age groups.

**Figure 2:**
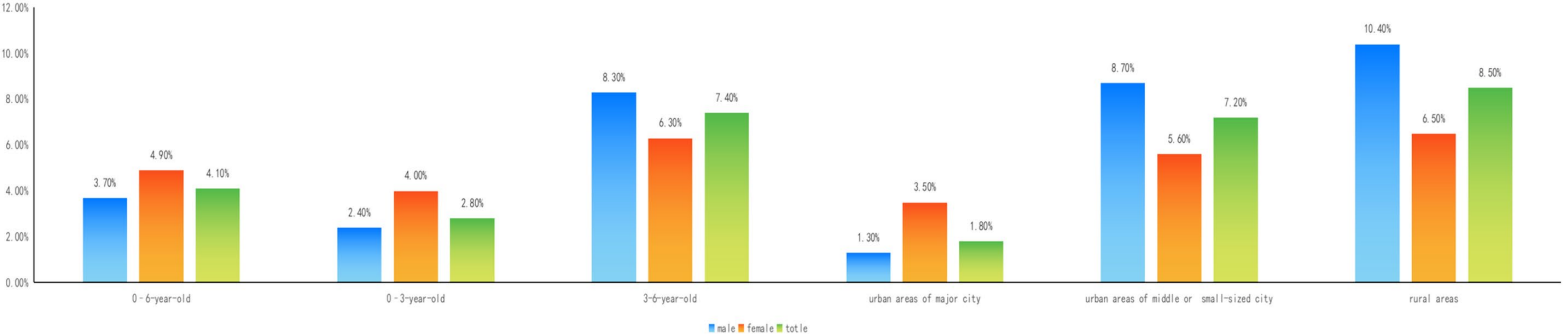
EBLL prevalence of children with different ages and genders in China.

In 0–3-Year-old children, female weighted prevalence of EBLL (4.0%) is higher than male (2.4%), while in 3–6-Year-old children, male (8.3%) is higher than female (6.3%). Among different areas, female EBLL weighted prevalence in urban area of major cities (3.5%) is higher, while male EBLL weighted prevalence in urban area of middle or small-sized city (8.7%) and in rural areas (10.4%) are higher (Table 2; Figure 2).

**Table 2:** EBLL prevalence of children with different ages and genders in China.

	Male	Female
*N* (%)	16,997 (52.2%)	15,546 (47.8%)
Weighted prevalence of EBLL	3.70%	4.90%
OR (95%CI)	Reference	1.36 (1.33,1.38)
*p* value		0
0–3-year-old		
*N* (%)	6,056 (52.1%)	5,571 (47.9%)
Weighted prevalence of EBLL	2.40%	4.00%
OR (95%CI)	Reference	1.68 (1.64,1.73)
*p* value		0
3–6-year-old		
*N* (%)	10,941 (52.3%)	9,975 (47.7%)
Weighted prevalence of EBLL	8.30%	6.30%
OR (95%CI)	Reference	0.75 (0.73,0.77)
*p* value		0
Urban areas of major city		
*N* (%)	5,364 (52.6%)	4,835 (47.4%)
Weighted prevalence of EBLL	1.30%	3.50%
OR (95%CI)	Reference	2.82 (2.71,2.92)
*p* value		0
Urban areas of middle or small-sized city		
*N* (%)	5,385 (52.1%)	4,941 (47.9%)
Weighted prevalence of EBLL	8.70%	5.60%
OR (95%CI)	Reference	0.62 (0.60,0.64)
*p* value		0
Rural areas		
*N* (%)	6,248 (52%)	5,770 (48%)
Weighted prevalence of EBLL	10.40%	6.50%
OR (95%CI)	Reference	0.60 (0.58,0.63)
*p* value		0

### 3.3 Proportion of EBLL in children by demographic characteristics

Table 3 shows the EBLL (BLL ≥50 μg/L) proportion of 0–6-year-old (from 1 month old to under 7 years old) children in China by demographic characteristics. A specific population of mothers with low educational levels (OR_illiterate_ = 6.43, OR_primary school_ = 4.55, OR_middle school_ = 3.46, OR_high school_ = 2.47), fathers (OR_illiterate_ = 8.28, OR_primary school_ = 6.99, OR_middle school_ = 4.86, OR_high school_ = 3.57), primary caregivers (OR_illiterate_ = 2.71, OR_primary school_ = 1.93, OR_middle school_ = 1.76) refering to the person who lived with the child and was mainly responsible for the child’s daily living arrangements, mother’s occupation worker (OR = 1.53) or peasantry (OR = 1.31), father’s (OR_worker_ = 1.24, OR_peasantry_ = 1.25), primary caregivers’ (OR_worker_ = 1.19, OR_peasantry_ = 1.23), low economic income (OR _<50,000 yuan_ = 3.3, OR_50,000–250,000 yuan_ = 2.11) is at risk of EBLL compared to other groups.

**Table 3:** Proportion of EBLL in children by demographic characteristics.

Demographic characteristics		*N* (%)	Proportion of EBLL	OR (95% CI)	*p* value
Maternal educational degree	Illiterate	269 (0.8%)	18.20%	6.43 (3.67, 11.3)	0
	Primary school	2,198 (6.8%)	13.60%	4.55 (2.8, 7.39)	0
	Middle school	12,528 (38.9%)	10.70%	3.46 (2.16, 5.55)	0
	High school	7,971 (24.8%)	7.90%	2.47 (1.53, 3.97)	0
	College/University	8,689 (27%)	4.60%	1.38 (0.85, 2.23)	0.19
	Higher education	538 (1.7%)	3.30%	Reference	
Paternal educational degree	Illiterate	99 (0.3%)	17.20%	8.28 (4.11, 16.7)	0
	Primary school	1,651 (5.2%)	14.90%	6.99 (4.3, 11.38)	0
	Middle school	12,063 (37.7%)	10.80%	4.86 (3.03, 7.78)	0
	High school	8,297 (25.9%)	8.20%	3.57 (2.22, 5.74)	0
	College/University	9,190 (28.7%)	4.90%	2.05 (1.27, 3.3)	0.003
	Higher education	737 (2.3%)	2.40%	Reference	
Caregiver’s educational degree	Illiterate	1,390 (4.5%)	14.70%	2.71 (2.11, 3.47)	0
	Primary school	4,713 (15.2%)	10.90%	1.93 (1.55, 2.4)	0
	Middle school	11,244 (36.2%)	10.10%	1.76 (1.43, 2.17)	0
	High school	7,064 (22.8%)	7.00%	1.18 (0.95, 1.47)	0.146
	College/University	4,925 (15.9%)	4.10%	0.68 (0.53, 0.87)	0.002
	higher education	1706 (5.5%)	6.00%	Reference	
Maternal occupation	Worker	2,382 (7.4%)	11.90%	1.53 (1.31, 1.79)	0
	Peasantry	6,342 (19.8%)	10.40%	1.31 (1.16, 1.48)	0
	Merchant	12,900 (40.3%)	8.20%	1.01 (0.91, 1.13)	0.814
	Technicians or administrator	4,425 (13.8%)	5.40%	0.64 (0.55, 0.75)	0
	Others or unknowing	5,970 (18.6%)	8.10%	Reference	
Paternal occupation	Worker	3,990 (12.5%)	10.60%	1.24 (1.09, 1.42)	0.001
	Peasantry	6,060 (19.1%)	10.60%	1.25 (1.11, 1.41)	0
	Merchant	8,574 (27%)	8.30%	0.95 (0.84, 1.06)	0.344
	Technicians or administrator	6,782 (21.3%)	5.70%	0.63 (0.55, 0.72)	0
	Others or unknowing	6,399 (20.1%)	8.70%	Reference	
Caregiver’s occupation	Worker	1798 (6.1%)	10.00%	1.19 (0.99, 1.42)	0.061
	Peasantry	6,982 (23.8%)	10.30%	1.23 (1.09, 1.38)	0.001
	Merchant	11,641 (39.7%)	8.30%	0.98 (0.87, 1.09)	0.663
	Technicians or administrator	2,821 (9.6%)	4.90%	0.55 (0.46, 0.67)	0
	Others or unknowing	6,065 (20.7%)	8.50%	Reference	
Annual household income (CNY)	<50,000 yuan	14,388 (45.7%)	10.70%	3.3 (2.51, 4.35)	0
	50,000–250,000 yuan	15,499 (49.3%)	7.10%	2.11 (1.6, 2.78)	0
	>250,000 yuan	1,579 (5%)	3.50%	Reference	

### 3.4 Risk factors of children’s behavior habits associated with EBLL

Information on children’s behavioral habits is also collected, including taking nutritional supplements, health habits, local customs, taking traditional Chinese medicine or folk prescriptions, and drinking water. Logistic regression analysis shows that risk factors of EBLL include unfrequently nail trimming (OR = 1.23, 1.35), sometimes/frequently hand-to-mouth activity (OR = 1.20, 1.39), frequently playing with dirt (OR = 1.37), contacting or burning tinfoil paper 3 times or more per year (OR = 1.24, 2.17), applying lead tetroxide (OR = 1.61), sometimes/frequent using traditional dinnerware such as tin pots (OR = 1.47, 1.85), using tap water, surface water, ground water (OR = 1.15, 1.60, 2.10). In contrast, protection factors include sometimes/frequently taking Zinc (OR = 0.75, 0.73), Iron (OR = 0.90, 0.83), Calcium (OR = 0.74, 0.61), Vitamin D supplements (OR = 0.72, 0.61), sometimes/frequently washing hands using soap or hand sanitizer before meals (OR = 0.76, 0.61).

After adjustment of gender, age, geographical region, annual household income, educational background and occupation of the parents and caregivers, risk factors concerning children’s behavior habits of EBLL include unfrequently nail trimming (OR = 1.14, 1.24), sometimes/frequently hand-to-mouth activity (OR = 1.23, 1.42), sometimes/frequently playing with dirt (OR = 1.12, 1.15), contacting or burning tinfoil paper 3 times or more per year (OR = 1.27, 1.96), applying lead tetroxide (OR = 1.56), sometimes/frequent using traditional dinnerware such as tin pots (OR = 1.34, 1.68), using folk prescriptions (OR = 1.23), using ground water (OR = 1.52). At the same time, sometimes/frequently taking Calcium (OR = 0.88, 0.82), Vitamin D supplements (OR = 0.86, 0.81), sometimes/frequently washing hands using soap or hand sanitizer before meals (OR = 0.87, 0.79) are protection factors (Table 4).

**Table 4:** Risk factors of children’s behavior habits associated with EBLL.

Factors of behavioral habits		*N* (%)	Proportion of EBLL	Crude OR (95% CI)	*p* value	Adjusted OR (95% CI)#	*p* value
Taking Zinc supplementation	Never	10,640 (33.4%)	10.10%	Reference		Reference	
	Sometimes	16,689 (52.4%)	7.70%	0.75 (0.69, 0.81)	0.00	0.86 (0.78, 0.94)	0
	Frequently	4,519 (14.2%)	7.60%	0.73 (0.65, 0.83)	0.00	0.88 (0.77, 1.02)	0.08
Taking Iron supplementation	Never	14,643 (46.3%)	9.00%	Reference		Reference	
	Sometimes	13,885 (43.9%)	8.10%	0.90 (0.83, 0.97)	0.01	0.99 (0.90, 1.08)	0.74
	Frequently	3,115 (9.8%)	7.60%	0.83 (0.72, 0.96)	0.01	0.95 (0.81, 1.12)	0.53
Taking Calcium supplementation	Never	4,596 (14.4%)	11.20%	Reference		Reference	
	Sometimes	17,574 (55.1%)	8.50%	0.74 (0.67, 0.83)	0.00	0.88 (0.79, 0.99)	0.04
	Frequently	9,751 (30.5%)	7.10%	0.61 (0.54, 0.69)	0.00	0.82 (0.71, 0.93)	0.00
Taking Vitamin D supplementation	Never	9,377 (29.4%)	10.70%	Reference		Reference	
	Sometimes	15,135 (47.5%)	7.90%	0.72 (0.66, 0.78)	0.00	0.86 (0.78, 0.95)	0.003
	Frequently	7,335 (23%)	6.80%	0.61 (0.55, 0.68)	0.00	0.81 (0.72, 0.93)	0.002
Washing hands using soap or hand sanitizer before meals	Rarely	14,215 (44.8%)	9.90%	Reference		Reference	
	Sometimes	12,613 (39.7%)	7.70%	0.76 (0.70, 0.83)	0.00	0.87 (0.79, 0.96)	0.01
	Frequently	4,915 (15.5%)	6.30%	0.61 (0.54, 0.69)	0.00	0.79 (0.69, 0.91)	0
Nail trimming	Less than every 2 weeks	19,320 (60.2%)	7.80%	Reference		Reference	
	Two weeks–one month	11,241 (35%)	9.40%	1.23 (1.14, 1.34)	0.00	1.14 (1.04, 1.25)	0
	More than 1 month	1,558 (4.9%)	10.20%	1.35 (1.13, 1.60)	0.00	1.24 (1.03, 1.50)	0.03
Hand-to-mouth activity	Rarely	14,315 (45.2%)	7.60%	Reference		Reference	
	Sometimes	13,736 (43.4%)	9.00%	1.20 (1.10, 1.31)	0.00	1.23 (1.12, 1.35)	0
	Frequently	3,596 (11.4%)	10.30%	1.39 (1.23, 1.57)	0.00	1.42 (1.24, 1.63)	0
Playing with dirt	Rarely	9,882 (31.2%)	7.90%	Reference		Reference	
	Sometimes	16,680 (52.7%)	8.20%	1.05 (0.95, 1.15)	0.34	1.12 (1.01, 1.24)	0.04
	Frequently	5,112 (16.1%)	10.50%	1.37 (1.22, 1.54)	0.00	1.15 (1.01, 1.32)	0.03
Contacting or burning tinfoil paper	0–1 times per year	28,156 (94.2%)	8.20%	Reference		Reference	
	2–3 times per year	1,234 (4.1%)	10.00%	1.24 (1.02, 1.50)	0.03	1.27 (1.03, 1.56)	0.03
	≥4 times per year	505 (1.7%)	16.20%	2.17 (1.70, 2.75)	0.00	1.96 (1.51, 2.56)	0
Applying lead tetroxide	Never	29,193 (98.5%)	8.50%	Reference		Reference	
	Yes	456 (1.5%)	12.90%	1.61 (1.22, 2.12)	0.00	1.56 (1.16, 2.09)	0
Using traditional dinnerware such as tinpots	Rarely	29,845 (94.4%)	8.30%	Reference		Reference	
	Sometimes	1,430 (4.5%)	11.70%	1.47 (1.25, 1.74)	0	1.34 (1.12, 1.61)	0
	Frequently	349 (1.1%)	14.30%	1.85 (1.37, 2.50)	0	1.68 (1.20, 2.35)	0
Using folk prescriptions	Never	28,721 (92.1%)	8.50%	Reference		Reference	
	Yes	2,454 (7.9%)	9.10%	1.09 (0.94, 1.25)	0.27	1.23 (1.05, 1.45)	0.01
Drinking water sources	Purified water	4,185 (13.4%)	6.80%	Reference		Reference	
	Tap water	22,464 (71.7%)	7.80%	1.15 (1.01, 1.31)	0.03	1.01 (0.88, 1.16)	0.91
	Surface water	591 (1.9%)	10.50%	1.60 (1.20, 2.14)	0	1.09 (0.78, 1.51)	0.63
	Ground water	4,097 (13.1%)	13.30%	2.10 (1.81, 2.45)	0	1.52 (1.29, 1.80)	0

# Adjusted by gender, age, parent’s and caregiver’s educational degree, parent’s and caregiver’s occupation and geographical region.

### 3.5 Risk factors of children’s living environment associated with EBLL

Logistic regression analysis shows that risk factors of children’s living environment associated with EBLL include living on the ground floor/The 2nd–3rd floor (OR = 1.93, 1.32), never/sometimes disusing first section of over night water (OR = 1.54, 1.35), frequently/sometimes passive smoking (OR = 1.32, 1.15), using coal or coal products/wood straw for cooking fuel (OR = 1.87, 1.41), using coal or coal products/wood straw for heating fuel (OR = 2.00, 1.55), none/chimney/window ventilated (OR = 1.60, 1.40, 1.28). There is no difference among houses of different distance to main road.

After adjustment of gender, age, geographical region, annual household income, educational background and occupation of the parents and caregivers, living on the ground floor (OR = 1.25), never/sometimes disusing first section of over night water (OR = 1.19, 1.16), frequently/sometimes passive smoking (OR = 1.24, 1.13), using coal or coal products for cocking fuel (OR = 1.50), using coal or coal products for heating fuel (OR = 1.68) remain risk factors of EBLL of 0–6-year-old children (Table 5).

**Table 5:** Risk factors of children’s living environment associated with EBLL.

Factors of children’s living environment		*N* (%)	Proportion of EBLL	Crude OR (95% CI)	*p* value	Adjusted OR (95% CI)#	*p* value
Floor of buildings	The ground floor	12,109 (37.9%)	11.00%	1.93 (1.74, 2.14)	0	1.25 (1.10, 1.42)	0
	The 2nd–3rd floor	10,402 (32.6%)	7.80%	1.32 (1.19, 1.48)	0	1.05 (0.93, 1.19)	0.43
	The 4th or above	9,419 (29.5%)	6.00%	Reference		Reference	
Disusing first section of over night water	Never	12,440 (39.3%)	9.50%	1.54 (1.36, 1.74)	0	1.19 (1.04, 1.37)	0.01
	Sometimes	13,519 (42.7%)	8.40%	1.35 (1.19, 1.52)	0	1.16 (1.02, 1.33)	0.03
	Frequently	5,665 (17.9%)	6.40%	Reference		Reference	
Passive smoking	Frequently	3,784 (12%)	10.10%	1.32 (1.17, 1.49)	0	1.24 (1.09, 1.41)	0
	Sometimes	11,469 (36.4%)	8.90%	1.15 (1.06, 1.25)	0	1.13 (1.03, 1.24)	0.01
	Never	16,229 (51.6%)	7.90%	Reference		Reference	
Cooking fuel	Coal or coal products	17,49 (5.5%)	13.70%	1.87 (1.62, 2.15)	0	1.50 (1.28, 1.76)	0
	Wood straw	3,187 (10%)	10.80%	1.41 (1.25, 1.60)	0	0.96 (0.84, 1.10)	0.59
	Clean energy	26,914 (84.5%)	7.90%	Reference		Reference	
Heating fuel	Coal or coal products	4,867 (16.5%)	13.40%	2.00 (1.81, 2.20)	0	1.68 (1.50, 1.88)	0
	Wood straw	1,506 (5.1%)	10.80%	1.55 (1.31, 1.84)	0	1.10 (0.91, 1.33)	0.31
	Clean energy	23,123 (78.4%)	7.20%	Reference		Reference	
Ventilation facilities	None	548 (1.7%)	11.30%	1.60 (1.22, 2.10)	0	1.02 (0.76, 1.36)	0.91
	Chimney	3,061 (9.7%)	10.00%	1.40 (1.22, 1.60)	0	0.91 (0.78, 1.06)	0.23
	Window ventilated	12,418 (39.6%)	9.20%	1.28 (1.17, 1.39)	0	1.01 (0.91, 1.11)	0.9
	Smoke exhaust ventilator	15,368 (49%)	7.40%	Reference		Reference	
Distance from house to main road	Along the street	4,813 (15.1%)	9.00%	1.09 (0.97, 1.22)	0.15	1.11 (0.98, 1.26)	0.09
	30–50 meters	3,238 (10.2%)	8.70%	1.05 (0.92, 1.2)	0.48	1.10 (0.95, 1.27)	0.2
	50–100 meters	7,271 (22.9%)	8.10%	0.97 (0.88, 1.07)	0.57	1.08 (0.97, 1.21)	0.15
	Above 100 meters	16,463 (51.8%)	8.40%	Reference		Reference	

# Adjusted by gender, age, parent’s and caregiver’s educational degree, parent’s and caregiver’s occupation and geographical region.

## 4 Discussion

Phasing out leaded petrol in 2000 and a series of environmental protection measures have dramatically decreased children’s BLL and the prevalence of lead poisoning in China over the past 20 years (28, 29); however, the CDC (2022) reported that there is no acceptable level of lead in the human body and that minimal lead levels can cause neurological damage to children (30). Lead exposure remains an important public health problem in China because of its extensive use in the manufacturing industry with rapid economic growth (20). This study assesses the prevalence and risk factors of EBLL in 0–6-year-old children in China. This group has more susceptibility to lead exposure for frequent hand-to-mouth activity and higher sensitivity to lead toxicity due to an immature nervous system.

The prevalence of EBLL, defined in this study as BLL ≥ 50 μg/L, among 0–6-year-old children in China is 4.1%. The Council of State and Territorial Epidemiologists’ (CSTE) blood lead reference value is 35 μg/L in the United States. This value is used to identify adults and children whose blood lead levels are higher than the 97.5th percentile of adults and children nationwide (32, 33).

This study explores the prevalence of EBLL in different geographic regions of China. The weighted prevalence of EBLL in the central region is much higher than the other two regions in China, which is consistent with the result that the pool rate of lead poisoning in China’s central region is higher than those in eastern and western regions as previous research showed (29). This change might be interpreted as the western region gradually reducing pollution industries with increasing awareness of environmental protection (34). The increasing support for primary medical and health care in western China from the central and local governments also contributes to the improving of children’s health. However, among provinces of western region, the children prevalence of EBLL in Qinghai Province is at a high level, especially the prevalence of EBLL in urban areas of major city (21.9%) and in urban areas of middle or small-sized city (16.2%), which both ranked first in their respective categories. The investigation sites of children in urban areas in Qinghai province are located in Xining City, Delingha and Geermu City, where coal resources are abundant, and the proportion of manufacturing and mining industries is rising year by year. Delingha and Geermu City belong to Mongolian and Tibetan Autonomous Region, where multi-ethnic population lives together. Environment pollution caused by industrial development, different national cultures and living habits bring a great challenge of the improvement of children’s living environment and health habits in such areas. Those results in the study suggest that we should pay more attention to monitoring children’s blood lead levels and strengthening health education in areas with different national culture and rapid industrial development.

This survey reveals the prevalence of children EBLL of rural areas in China is much higher than of urban areas, which suggests that children in rural areas is at high risk of lead exposure. Considering geographic factor, children in rural areas of eastern and western regions is at high risk of EBLL, while children in urban areas of middle or small-sized city in central region should be pay more attention of possible lead exposure.

Some studies reveal the association between controlling environmental lead pollution in the atmosphere and considerable and sustained reduction of a population’s blood lead level (16, 35). We found that prevalence of EBLL differed significantly by province with the highest in Hebei province (18.5%), which dominated by industry with proportion accounts for 53.5%, especially the iron and steel industry as the first pillar industry in Hebei Province in 2011. Among different districts, weighted prevalence of EBLL in rural area of Hebei province, where mineral resources such as coal and coal bed methane were abundant, is high up to 41.0%. This result suggests that we should pay attention to screening the blood lead levels of children in mineral rich areas.

In this study, we observed weighted prevalence of children with BLL ≥50 μg/L (EBLL) is higher in female than in male, which seems inconsistent with previous results with higher proportion of BLL ≥50 μg/L in male than in female (31). However, in consideration of multi-stage sampling method of the survey, the proportion does not represent prevalence. In order to make the sample better reflect the overall characteristics, weighting issue should be taken into account when prevalence is mainly discussed in this paper. Though boys has higher BLLs compared to girls as previous results, the prevalence of girls with BLL ≥50 μg/L is higher. When subgroups are discussed, the situation varies among different age groups and area groups. In 0–3-Year-old children, female weighted prevalence of EBLL is higher than male, while in 3–6-Year-old children, male is higher than female. Also, in major city districts, female weighted prevalence of EBLL is higher than male, while in middle or small-sized city districts and in rural areas, the situation is on the contrary. The same phenomenon has been reported about age-dependent manner of EBLL prevalence increasing with age growth (36). In this survey, EBLL prevalence is higher in 3–6-year-old children, and BLL is also higher compared to 0–3-year-old children which published in our previous article (31). More boys’ bad habits and outdoor activity in 3–6-year-old children or in rural areas are likely to increase the risk of lead exposure (18, 35, 37).

As far as demographic characteristics are concerned, this study shows that mothers with low educational levels, fathers, caregivers, low socioeconomic family income and mother’s occupation worker or peasantry, fathers, caregivers seemed to have a higher prevalence of EBLL. However, the trends of both BLL and EBLL prevalence increased significantly in an age-dependent manner was not observed in this study, which was needed further research and exploration. As for family socioeconomic status, parents or caregivers with higher educational levels are better aware of avoiding lead exposure and promoting children’s good hygiene habits, reducing opportunities for children suffering from lead poisoning (26). Children living in poverty are more likely to reside in older houses with lead-based paint and lead-containing plumbing and have a higher likelihood of having nutritional imbalances such as iron deficiency (38).

This study examined risk factors related to children’s behavioral tendency toward EBLL. The results show that unfrequently nail trimming, sometimes/frequently hand-to-mouth activity, sometimes/frequently playing with dirt were the risk factors of EBLL, as previous research has shown (39–43).

In this study we also explored local traditional custom and folk prescription impacting on EBLL incidence. We found that contacting or burning tinfoil paper 3 times or more per year, sometimes/frequent using traditional dinnerware such as tin pots, applying lead tetroxide, using folk prescriptions were risk factors of children EBLL. Local customs and traditions (such as contacting or burning tinfoil paper several times or using tin pots as dinnerware) might be related to lead exposure. Tinfoil paper is a complex of tin and lead with a surface layer of metal-containing lead. According to local customs in East China, during funerals, people often fold or burn tinfoil paper at home, and lead on the surface layer releases polluting indoor air, which might result in lead exposure (44). Another study showed that tinfoil paper manufacture in family workshops seriously impacts the BLLs of operators and their family members (45). We also found that tin pots, a traditional vessel containing lead, may cause EBLL if children use them (46). Traditional Chinese medicine believes that the rational use of lead can treat various difficult and complicated diseases, but modern traditional Chinese medicine rarely uses it. Lead poisoning caused by lead containing drugs often comes from folk remedies. Lead tetroxide (chemical formula Pb3O4) is popular in some remote rural areas of southern China (47) for treating skin diseases, such as prickly heat and eczema; it is typically applied on children’s necks, armpits, and groin areas. A case report covered that a 3-year-old boy with BLL 303 μg/L and his 6-month-old sister with BLL 385 μg/L had used lead tetroxide instead of baby power (21). An investigation of approximately 222 children living in a Chinese rural area showed that lead-containing powder use is significantly associated with EBLL (47). Other folk remedies that are significantly correlated with higher BLL are also reported, such as folk remedies for treating epilepsy (48–50). Although the medicinal value of lead was recorded in the earliest Chinese pharmacological works, Shennong Classic of Materia Medica, for example, adding minerals such as lead to enhance the stability, sustainability, efficacy and storability of herbal medicines, modern Chinese medicine rarely used lead. Many studies revealed that the use of lead-containing herbs or lead-containing preparations mostly originated from folk remedies and long-term use of such lead-containing drugs may suffer from high risk of elevated blood lead levels or even lead poisoning (22, 45, 47, 51).

Furthermore, this study found that decentralized water supply was one of the risk factors for increased blood lead levels in children. In rural areas of China, decentralized water supply projects primarily utilized shallow well, mostly by household construction and management, and with the lack of water quality testing and monitoring. The findings show that the ground water with a non-central water supply was unsafe for lead exposure. Contamination of soil, water (groundwater and surface water), sediment, and air with hazardous and toxic compounds is one of the most severe and challenging problems that the industrialized world faces (52). A cross-sectional survey in Pakistan was conducted using water samples from drinking water sources. Of the 216 ground and surface water samples collected, 86% had lead levels higher than the World Health Organization’s maximum acceptable concentration of 10 ppb. The mean lead concentration in groundwater was 146 ppb, significantly higher than in surface water, with a lead concentration of 77.1 ppb (53).

Previous studies also found that elevated lead concentration in water often occurs due to corrosive water effects on pipeline materials, such as plumbing, coating, solder, pipes, pipe joints, and fittings (54). That might partially explain the result in this study that never/sometimes disusing first section of over night water might increase risk of EBLL.

This study also discussed children’s living environmental risk factors associated with EBLL. Lead pollution mainly comes from dust and sediment on the ground or suspended in the air. These are released by industrial metal smelting, mining enterprises, and related industrial and manufacturing industries, such as battery manufacturing, printing, mechanical manufacturing, and shipbuilding (18, 55). The results of a meta-analysis show that the corrected pooled lead poisoning rates of populations living around mining areas (70%) and industrial areas (57.5%) are much higher than those in urban areas (9.6%), suburban areas (23.6%), and rural areas (23.8%) (29). The total suspended lead particle counts in ambient air and lead concentrations are ten times higher than in the upper air above the ground where children breathe (40).

Our investigation suggested that the parents’ occupational exposure to lead is a risk factor for EBLL among children. Lead dust from parents’ surfaces and work clothing can be digested or inhaled by children at home (37). Parents may also carry the lead from the workplace to home in their clothing.

We find that living on the ground might increase the EBLL risk due to contacting dust, soil, or smoke. Lead in the air and the soil can enter the room of lower floors more easily than on higher floors, which leads to a higher rate of EBLL occurrence. A study tested that reducing 1,000 ppm or more lead in soil accessible to children would decrease at least 30 μg in BLLs. The results demonstrated that lead-contaminated soil or dust contributes to the lead burden in children (56).

We also verify that passive smoking as a risk factor is associated with EBLL, which is similar to the result of a previous study (37). Cigarettes contain heavy metals, such as cadmium and lead, and second-hand smoking exposure can increase the level of lead in children’s blood (26, 57, 58). It is estimated that 72% of non-smokers, including 180 million children under the age of 15, who live in China are exposed to second-hand smoke (59, 60).

This study also focuses on the impact of indoor air pollution from coal burning on children EBLL. During the coal combustion process, various atmospheric pollutants can be generated, including the heavy metal element lead, which is highly volatile. The result revealed the harmfulness of indoor cooking and heating fueled by coal or coal products. Therefore, coal which is commonly used as indoor fuel in northern China, is not recommended as a household fuel to avoid lead exposure.

Unlike previous research results, we did not find an increase in the prevalence of EBLL with a decrease in the distance between the residence and the main road. Local researches in China have found that after the implementation of unleaded gasoline, automobile exhaust is no longer the main source of atmospheric lead pollution. Lead pollution in road dust is mainly controlled by coal combustion emissions and non-ferrous metallurgical emissions (61, 62). Thus, the distance between the residence and the main road is not a risk factor of EBLL in China.

The strength of this study is its reporting prevalence of EBLL in Chinese children aged 0–6 years (from 1 month old to under 7 years old) in a large sample size and exploring risk factors for children lead exposure, especially in areas of traditional Chinese customs and the use of folk remedies. These issues have been rarely addressed in previous studies. Nonetheless, this study has some limitations. While adequate for assessing the BLLs of most Chinese children aged 0–6 years, the sample size may not be sufficient to reflect the extent of overall risk associated with lead exposure. Furthermore, this study did not directly measure the lead level of pollution sauces, such as the atmosphere, drinking water, soil, house dust, diet, and others in the children’s proximate environment. Moreover, as a cross-sectional study, the data reflect children’s lead exposure over the study period.

## 5 Conclusion

This study reveals the prevalence and risk factors for EBLL (BLL ≥ 50 μg/L) in 0–6-year-old children (from 1 month old to under 7 years old) in China. The pediatric population with high-risk factors should be given attention of lifestyle habits and living environment of lead exposure. This study also indicates that lead poisoning in children is preventable by removing high-risk factors and promoting protective factors. We hope this study will help public health education and inform policy for preventing and eradicating lead poisoning in China.

## Data Availability

The data are available from the corresponding author on reasonable request.
